# Impaired Endothelium-Dependent Vasodilation and Increased Levels of Soluble Fms-like Tyrosine Kinase-1 Induced by Reduced Uterine Perfusion Pressure in Pregnant Rats: Evidence of Protective Effects with Sodium Nitrite Treatment in Preeclampsia

**DOI:** 10.3390/ijms252011051

**Published:** 2024-10-15

**Authors:** Maria Luiza Santos Da Silva, Sáskia Estela Biasotti Gomes, Laisla Zanetoni Martins, Serginara David Rodrigues, Cristal de Jesus Toghi, Carlos Alan Dias-Junior

**Affiliations:** Department of Biophysics and Pharmacology, Institute of Biosciences, Sao Paulo State University (UNESP), Botucatu 18618-689, SP, Brazil; mls.silva@unesp.br (M.L.S.D.S.); saskia.estela@unesp.br (S.E.B.G.); laisla.martins@unesp.br (L.Z.M.); serginara.rodrigues@unesp.br (S.D.R.); c.toghi@unesp.br (C.d.J.T.)

**Keywords:** preeclampsia, nitric oxide, sodium nitrite, placental ischemia, endothelial dysfunction

## Abstract

Preeclampsia (PE) is a hypertensive disorder of pregnancy and is associated with increases in soluble fms-like tyrosine kinase-1 (sFlt-1) and reductions in nitric oxide (NO) levels. Placental ischemia and hypoxia are hypothesized as initial pathophysiological events of PE. Nitrite (NO metabolite) may be recycled back to NO in ischemic and hypoxic tissues. Therefore, this study examined the sodium nitrite effects in an experimental model of PE. Pregnant rats received saline (Preg group) or sodium nitrite (Preg + Na-Nitrite group). Pregnant rats submitted to the placental ischemia received saline (RUPP group) or sodium nitrite (RUPP + Na-Nitrite group). Blood pressure, placental and fetal weights, and the number of pups were recorded. Plasma levels of NO metabolites and sFlt-1 were also determined. Vascular and endothelial functions were also measured. Blood pressure, placental and fetal weights, the number of pups, NO metabolites, sFlt-1 levels, vascular contraction, and endothelium-dependent vasodilation in the RUPP + Na-Nitrite rats were brought to levels comparable to those in Preg rats. In conclusion, sodium nitrite may counteract the reductions in NO and increases in sFlt-1 levels induced by the placental ischemia model of PE, thus suggesting that increased blood pressure and vascular and endothelial dysfunctions may be attenuated by sodium nitrite-derived NO.

## 1. Introduction

Preeclampsia (PE) is a severe hypertensive disorder of pregnancy and is related to a large percentage of maternal–fetal mortality and morbidity [1]. Annually, around 4 million pregnant women are diagnosed with PE worldwide, being reported as the main cause of death for more than 70,000 women and 500,000 babies [2]. After the occurrence of PE, there are also potential risks and negative consequences for mothers and children later in life, which are manifested as cardiovascular diseases [3].

Regarding the pathophysiology of PE, it has been suggested that trophoblasts’ invasion and remodeling of the uterine spiral arteries are impaired and may be initial events prior to the deficient placentation, which are followed by placental ischemia/hypoxia [4]. The initial signs of PE in pregnant women are often unclear, and one or more symptoms may occur [5]. The diagnosis is confirmed when there are increases in systolic/diastolic blood pressure greater than or equal to 140/90 mmHg as well as proteinuria with the presence of albumin in urine, which is strongly related to kidney damage [6]. In the final stage of the PE, fetal prematurity and fatal cases have been related to the occurrence of eclampsia [7].

The clinical manifestations of PE have been related to maternal endothelial dysfunction that may be the result from the angiogenic imbalance, which is biochemically determined by angiogenic and antiangiogenic factors present in the plasma of women with PE [8]. Exacerbated increases in circulating sFlt-1 followed by decreases in angiogenic factors such as vascular endothelial growth factor (VEGF) and placental growth factor (PlGF) are biochemical markers found in the maternal plasma of PE [9]. sFlt-1 or VEGF Flt-1 acts as an important molecule produced by trophoblasts that is released into maternal blood circulation [10,11]. The lack of cytoplasmic and transmembrane domains causes the sFlt-1 to behave as a soluble receptor that binds to circulating VEGF and PlGF, i.e., sFlt-1 acts as a potent antiangiogenic molecule [12,13]. However, there is still no effective treatment to attenuate the exacerbated increases in circulating sFlt-1 in maternal plasma of PE [14]. In addition, previous studies have shown that increases in s-Flt-1 in plasma are inversely associated with decreases in endogenous NO formation in PE [14,15,16,17].

Experimental evidence demonstrates that NO is an important mediator of vasodilation and it may also modulate trophoblastic invasion and mediate the remodeling of uterine spiral arteries to adequately supply the developing fetus [18]. Hence, a potential alternative would be restoring NO levels independently of the signaling pathway related to endothelial NO synthase (eNOS), which is impaired in PE and is associated with endothelial dysfunction [19]. Recently, it has been demonstrated that nitrite (NO metabolite) may be recycled back to NO, and this recycling may occur especially in ischemic tissues [20].

Therefore, in the present study, placental ischemia in pregnant rats was induced by reduced uteroplacental perfusion pressure (RUPP), which is a model of PE [21]. The RUPP model in rats is characterized by increases in blood pressure and reductions in fetal and placental weights [22]. However, no previous study has yet examined the effects of sodium nitrite treatment in increased circulating s-Flt-1 in the RUPP model of PE. Our main hypothesis is that sodium nitrite treatment attenuates increases in maternal blood pressure, protects against fetal and placental growth restrictions, mitigates the exacerbated increases in circulating s-Flt-1, and attenuates maternal endothelial dysfunction.

## 2. Results

### 2.1. Effects of Sodium Nitrite Treatment on Maternal Blood Pressure

Maternal systolic blood pressure (SBP) was monitored on pregnancy days 11 and 13, and there were no differences on pregnancy days preceding the surgical procedure to the induction of PE on pregnancy day 14.

**Figure 1:**
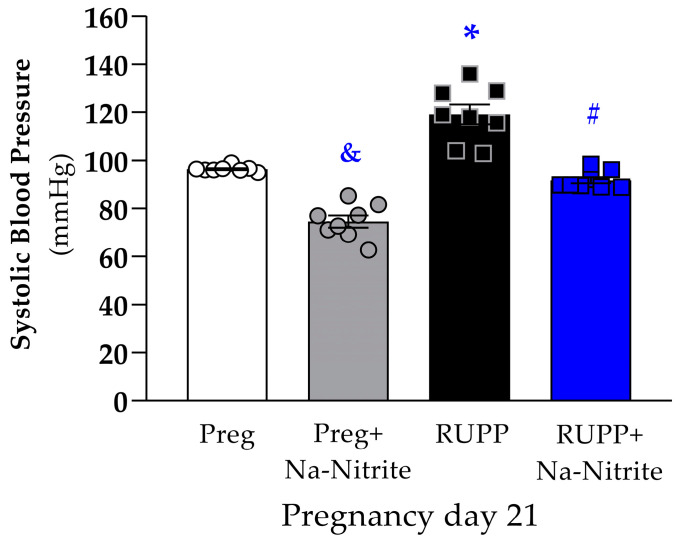
Effects of sodium nitrite on systolic blood pressure (SBP) measured on pregnancy day 21 in the Preg, Preg+Na-Nitrite, RUPP, and RUPP+Na-Nitrite groups (*n* = 8–10 animals per group). Values represent the mean ± SEM. ^&^ *p* < 0.05 vs. the Preg, RUPP, and RUPP+Na-Nitrite groups; * *p* < 0.05 vs. the Preg group; ^#^ *p* < 0.05 vs. the RUPP group.

On pregnancy day 21, SBP was significantly higher in RUPP rats compared to the Preg group (119 ± 4 vs. 96 ± 1 mmHg; Figure 1), whereas no differences were observed between the RUPP+Na-Nitrite and Preg groups (92 ± 2 vs. 96 ± 1 mmHg; Figure 1). Moreover, SBP was statistically lower in Preg+Na-Nitrite rats compared to the Preg, RUPP, and RUPP+Na-Nitrite groups (75 ± 3 vs. 96 ± 1, 119 ± 4, and 92 ± 2 mmHg, respectively; Figure 1).

### 2.2. Effects of Sodium Nitrite Treatment on Fetal and Placental Parameters

**Figure 2:**
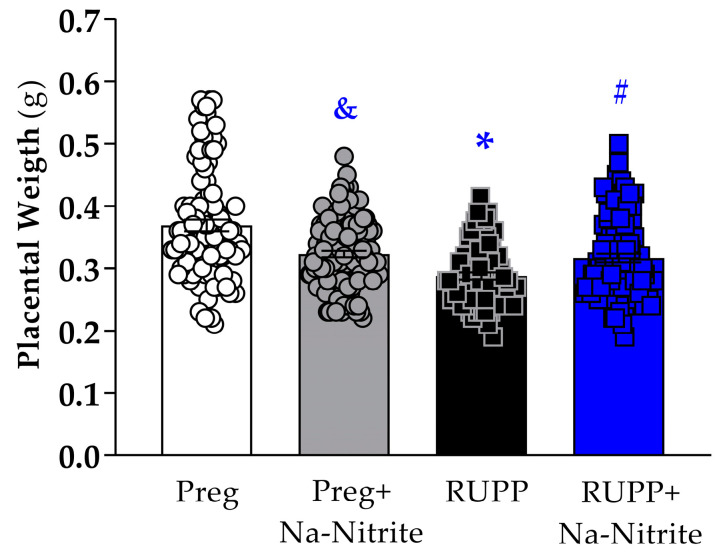
Effects of sodium nitrite on placental weight recorded in the Preg, Preg+Na-Nitrite, RUPP, and RUPP+Na-Nitrite groups (*n* = 8–10 mothers per group). Values represent the mean ± SEM. ^&^ *p* < 0.05 vs. the Preg and RUPP groups; * *p* < 0.05 vs. the Preg group; ^#^ *p* < 0.05 vs. the RUPP group.

Placental weight was significantly reduced in the RUPP group compared to Preg rats (0.28 ± 0.005 vs. 0.36 ± 0.009 g; Figure 2), whereas no difference was observed between the RUPP+Na-Nitrite and Preg groups (0.31 ± 0.006 vs. 0.36 ± 0.009 g, respectively; Figure 2). Moreover, placental weight was statistically lower in Preg+Na-Nitrite rats compared to the Preg group (0.32 ± 0.005 vs. 0.36 ± 0.009 g; Figure 2). Also, placenta weight was statistically higher in the Preg+Na-Nitrite group compared to the RUPP group (0.32 ± 0.005 vs. 0.28 ± 0.005 g; Figure 2).

**Figure 3:**
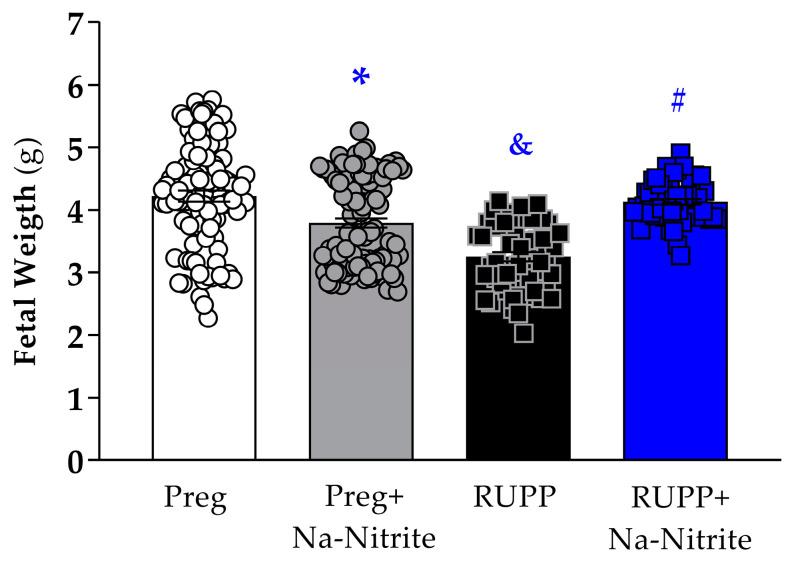
Effects of sodium nitrite on fetal weight recorded in the Preg, Preg+Na-Nitrite, RUPP, and RUPP+Na-Nitrite groups (*n* = 8–10 mothers per group). Values represent the mean ± SEM. * *p* < 0.05 vs. the Preg group; ^&^ *p* < 0.05 vs. the Preg and Preg+Na-Nitrite groups; ^#^ *p* < 0.05 vs. the RUPP group.

Fetal weight was significantly reduced in the RUPP group compared to Preg rats (3.25 ± 0.07 vs. 4.25 ± 0.08 g; Figure 3), whereas no difference was observed between the RUPP+Na-Nitrite and Preg groups (4.09 ± 0.05 vs. 4.25 ± 0.08 g; Figure 3). Moreover, fetal weight was statistically lower in Preg+Na-Nitrite rats compared to the Preg group (3.79 ± 0.07 vs. 4.25 ± 0.08 g; Figure 3). Also, fetal weight was statistically higher in the Preg+Na-Nitrite group compared to the RUPP group (3.79 ± 0.07 vs. 3.25 ± 0.07 g; Figure 3).

**Figure 4:**
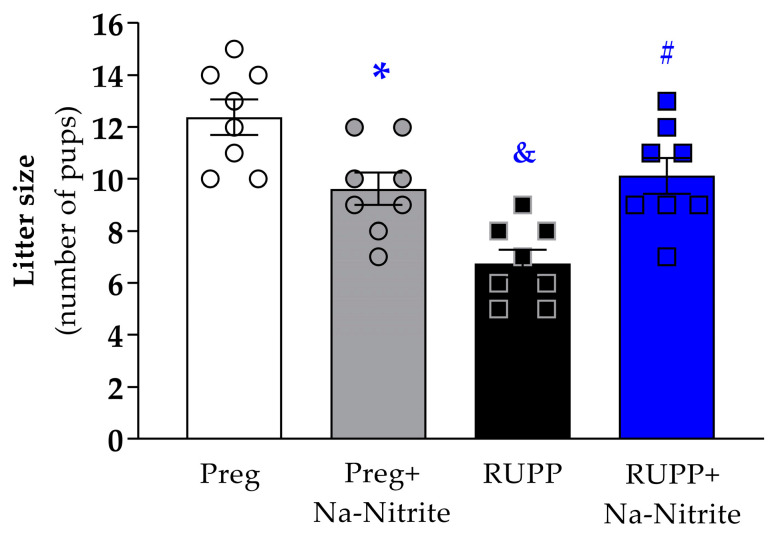
Effects of sodium nitrite on litter size (total number of pups) noted in the Preg, Preg+Na-Nitrite, RUPP, and RUPP+Na-Nitrite groups (*n* = 8–10 mothers per group). Values represent the mean ± SEM. * *p* < 0.05 vs. the Preg group; ^&^ *p* < 0.05 vs. the Preg and Preg+Na-Nitrite groups; ^#^ *p* < 0.05 vs. the RUPP group.

The number of pups per litter was significantly reduced in the RUPP group compared to Preg rats (6.7 ± 0.5 vs. 12.3 ± 0.6; Figure 4), whereas no difference was observed between the RUPP+Na-Nitrite and Preg groups (10.1 ± 0.6 vs. 12.3 ± 0.6; Figure 4). Moreover, the number of pups per litter was statistically lower in Preg+Na-Nitrite rats compared to the Preg group (9.6 ± 0.6 vs. 12.3 ± 0.6; Figure 4). Also, the number of pups was statistically higher in the Preg+Na-Nitrite group compared to the RUPP group (9.6 ± 0.6 vs. 6.7 ± 0.5; Figure 4).

### 2.3. Effects of Sodium Nitrite Treatment on Plasmatic NO Metabolites (NOx)

**Figure 5:**
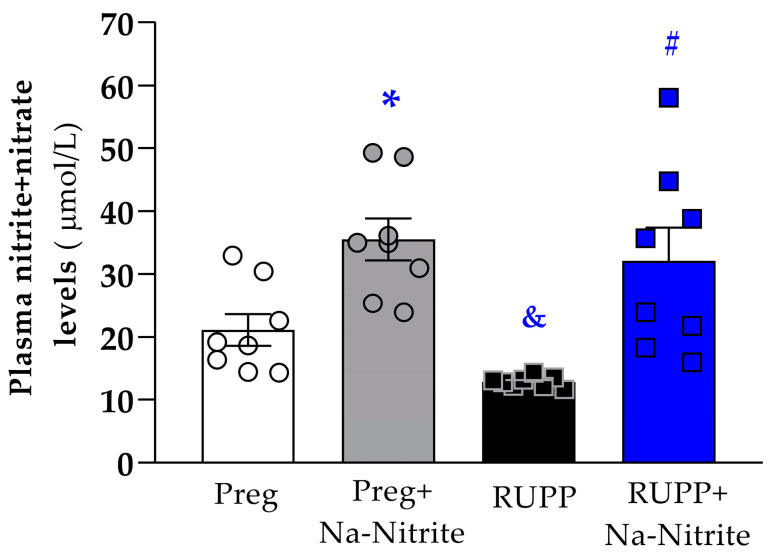
Effects of sodium nitrite on plasmatic NO metabolites (nitrite+nitrate levels) in the Preg, Preg+Na-Nitrite, RUPP, and RUPP+Na-Nitrite groups (*n* = 8–10 animals per group). Values represent the mean ± SEM. * *p* < 0.05 vs. the Preg group; ^&^ *p* < 0.05 vs. the Preg and Preg+Na-Nitrite groups; ^#^ *p* < 0.05 vs. the RUPP group.

Plasmatic NO metabolites were significantly lower in RUPP rats compared to the Preg group (12 ± 1 vs. 21 ± 3 µmol/L; Figure 5). However, higher plasmatic NO metabolites were observed in RUPP+Nitrite rats compared to the RUPP group (31 ± 5 vs. 12 ± 1 µmol/L; Figure 5). Moreover, higher plasmatic NO metabolites were observed in Preg+Na-Nitrite rats compared to Preg and RUPP rats (35 ± 3 vs. 21 ± 3 and 12 ± 1, respectively; Figure 5).

### 2.4. Effects of Sodium Nitrite Treatment on Circulating sFlt-1 Levels

**Figure 6:**
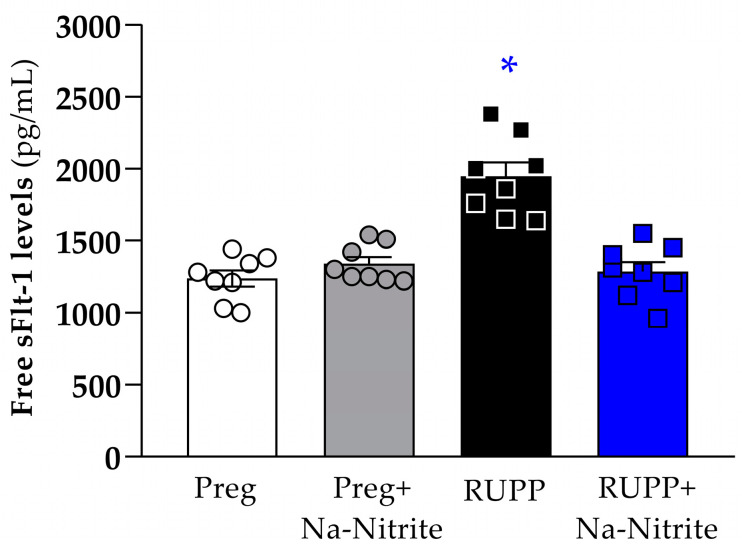
Effects of sodium nitrite on circulating sFlt-1 levels in plasma from the Preg, Preg+Na-Nitrite, RUPP, and RUPP+Na-Nitrite groups (*n* = 8–10 animals per group). Values represent the mean ± SEM. * *p* < 0.05 vs. the Preg, Preg+Na-Nitrite, and RUPP+Na-Nitrite groups.

Plasma levels of sFlt-1 were significantly higher in RUPP rats compared to the Preg, Preg+Na-Nitrite, and RUPP+Na-Nitrite groups (1948 ± 96 vs. 1238 ± 56, 1340 ± 47, and 1285 ± 67 pg/mL, respectively; Figure 6). Moreover, there were no statistical differences among other groups.

### 2.5. Effects of Sodium Nitrite on Phenylephrine-Induced Contraction in Aortic Rings

**Figure 7:**
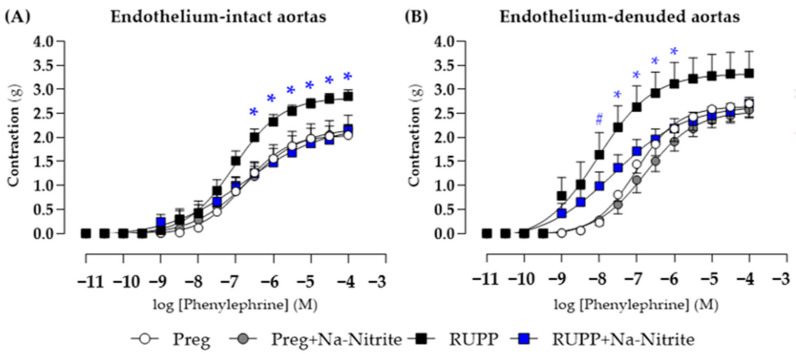
Effects of sodium nitrite on the vascular reactivity of the thoracic aorta rings in the contraction induced by phenylephrine with (*n* = 2 rings per animal, (**A**)) or without (*n* = 2 rings per animal, (**B**)) endothelium in the Preg, Preg+Na-Nitrite, RUPP, and RUPP+Na-Nitrite groups (*n* = 8–10 animals per group). Values represent the mean ± SEM. * *p* < 0.05 vs. the Preg, Preg+Na-Nitrite, and RUPP+Na-Nitrite groups; ^#^ *p* < 0.05 for the RUPP and RUPP+Na-Nitrite vs. the Preg and Preg+Na-Nitrite groups.

In endothelium-intact thoracic aortic rings, phenylephrine-induced contraction was greater in the RUPP group in the concentration range of 10^−7.5^–10^−4^ M compared with the Preg, Preg+Na-Nitrite, and RUPP+Na-Nitrite groups (Figure 7A and Table S1), whereas no differences were observed among other groups. Moreover, maximal phenylephrine-induced contraction was greater in the RUPP group compared with the Preg, Preg+Na-Nitrite, and RUPP+Na-Nitrite groups (2.85 ± 0.13 vs. 2.04 ± 0.09, 2.17 ± 0.08, and 2.16 ± 0.11, respectively; Figure 7A and Table S1).

In endothelium-removed thoracic aortic rings, phenylephrine-induced contraction was greater in the RUPP and RUPP+Na-Nitrite groups in the concentration of 10^−8^ M when compared with the Preg and Preg+Na-Nitrite groups (1.64 ± 0.45 and 0.98 ± 0.15 vs. 0.22 ± 0.08 and 0.27 ± 0.10 g, respectively; Figure 7B and Table S1). In the concentration range of 10^−7.5^ to 10^−6^ M, phenylephrine-induced contraction was greater in the RUPP group compared with the Preg, Preg+Na-Nitrite, and RUPP+Na-Nitrite groups (Figure 7B and Table S1). Moreover, maximal phenylephrine-induced contraction was greater in the RUPP group compared with the Preg, Preg+Na-Nitrite, and RUPP+Na-Nitrite groups (3.33 ± 0.45 vs. 2.53 ± 0.19, 2.17 ± 0.10, and 2.49 ± 0.15 g, respectively; Figure 7B and Table S1).

### 2.6. Effects of Sodium Nitrite on Acetylcholine-Induced Relaxation in Aortic Rings

**Figure 8:**
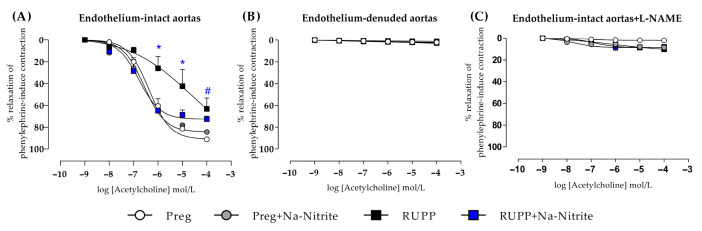
Effects of sodium nitrite on the vascular reactivity of acetylcholine-induced relaxations in the endothelium-intact thoracic aortas (*n* = 2 rings per animal, (**A**)) or endothelium-denuded thoracic aortas (*n* = 2 rings per animal, (**B**)) or endothelium intact thoracic aortas pre-incubated with L-NAME (*n* = 2 rings per animal, (**C**)) in the Preg, Preg+Na-Nitrite, RUPP, and RUPP+Na-Nitrite groups (*n* = 8–10 animals per group). Values represent the mean ± SEM. * *p* < 0.05 vs. the Preg, Preg+Na-Nitrite, and RUPP+Na-Nitrite groups; ^#^ *p* < 0.05 vs. the Preg group.

In endothelium-intact thoracic aortic rings, acetylcholine-induced relaxation was impaired in the RUPP in the concentration range of 10^−6^–10^−5^ M compared with the Preg, Preg+Na-Nitrite, and RUPP+Na-Nitrite groups (Figure 8A and Table S1). In the concentration of 10^−4^, acetylcholine-induced relaxation was significantly impaired in RUPP compared with the Preg group, but the RUPP was not statistically different from the Preg+Na-Nitrite and RUPP+Na-Nitrite groups (Figure 8A and Table S1). Moreover, maximal acetylcholine-induced relaxation was impaired in the RUPP group compared with the Preg group (64 ± 9 vs. 91 ± 16; Figure 8A and Table S1).

Endothelium-denuded thoracic aortic rings showed no differences among all groups, demonstrating that the mechanical removal of the endothelium abolished the acetylcholine-induced relaxation (Figure 8B and Table S1).

In endothelium-intact thoracic aortic rings pre-incubated with L-NAME, no significant relaxation was observed in aortic rings from all groups, demonstrating that the acetylcholine-induced relaxation was pharmacologically inhibited (Figure 8C and Table S1).

### 2.7. Effects of Sodium Nitrite on Endothelium-Independent Relaxation in Aortic Rings

**Figure 9:**
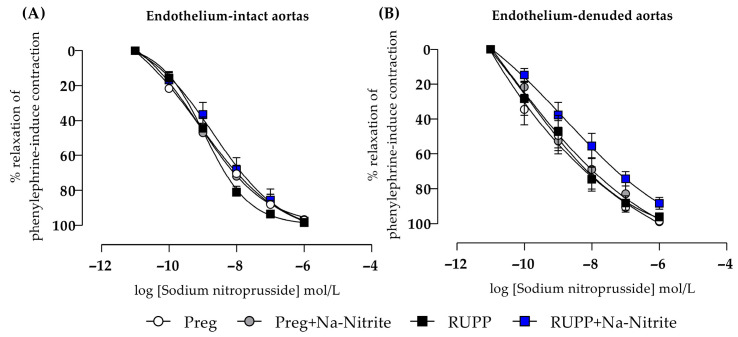
Effects of sodium nitrite on the vascular reactivity of sodium nitroprusside-induced relaxations in the endothelium-intact thoracic aortas (*n* = 2 rings per animal, (**A**)) or endothelium-denuded thoracic aortas (*n* = 2 rings per animal, (**B**)) from the Preg, Preg+Na-Nitrite, RUPP, and RUPP+Na-Nitrite groups (*n* = 8–10 animals per group). Values represent the mean ± SEM.

In endothelium-intact thoracic aortic rings, sodium nitroprusside-induced relaxation showed no statistical differences among all groups (Figure 9A and Table S1). In endothelium-removed thoracic aortic rings, no statistical differences were also observed among all groups (Figure 9B and Table S1).

## 3. Discussion

Excess sFlt-1 and reduced NO bioavailability in PE have been associated with increases in blood pressure, the dysfunction of the maternal endothelium, and the growth restriction of the fetus and placenta [23]. Placental ischemia has been hypothesized as one of the main etiological factors, and it is associated with increased sFlt-1 levels in the maternal circulation of pregnancies complicated by PE, but conclusive confirmation for this hypothesis is still lacking [24]. To the best of our knowledge, this is the first evidence demonstrating that it may be possible to reduce the systemic sFlt-1 levels through treatment with sodium nitrite-derived NO in a placental ischemia-induced model of PE in rats.

In the present study, the experimental model of PE in rats was able to mimic the pathophysiological features found in preeclamptic women [25,26,27]. Our findings refer to a significant increase in maternal blood pressure, the weight loss of placenta and fetus, and a reduced litter size. In addition, we found decreases in plasmatic NO metabolites and increases in circulating sFlt-1 levels. Furthermore, our current results show increased vasoconstriction-induced phenylephrine and impaired acetylcholine-induced vasodilation in the aortas of pregnant rats submitted to the placental ischemia-induced preeclamptic model, thus demonstrating that vascular and endothelial dysfunctions were induced experimentally by placental ischemia, which corroborate the findings previously reported in women [28] and pregnant rats [29] with PE [30,31].

Recent evidence has demonstrated that a NO metabolite named nitrite may be recycled back into the NO, which may restore the circulating NO levels independently of endothelial NO synthase [32]. The reduction of nitrite in NO may be enhanced in hypoxic and ischemic tissues, conductions in which oxygen (substrate to the NO synthesis by endothelial NO synthase) is impaired, thus representing an alternative pathway to generate the NO during ischemia [33]. In the present study, increases in circulating NO metabolites were found in pregnant rats submitted to placental ischemia and treated with sodium nitrite. Moreover, sodium nitrite treatment attenuated all other adverse outcomes induced by experimental PE in rats, in which measurements of blood pressure, fetal and placental weights, litter size, circulating sFlt-1, vascular contraction, and endothelium-dependent relaxation in the RUPP+Na-Nitrite rats were brought to levels comparable to those in control Preg rats.

It has been experimentally demonstrated in vitro that the production and accumulation of sFtl-1 in placental trophoblasts may be regulated by NO under hypoxia conditions [34]. Although the etiology of PE is still unclear, the inadequate invasion of trophoblasts into uterine spiral arteries early in pregnancy has been suggested as an initial event to the development of ischemia followed by intermittent blood flow in the uteroplacental circulation, which contributes to the placental hypoxia later in pregnancy [35]. It is known that trophoblast cells from preeclamptic placentas produce more sFtl-1 than those from healthy placentas [10], and this may be mediated in part by placental hypoxia [36,37,38,39]. Our present results suggest that sodium nitrite-derived NO significantly prevented increases in sFtl-1 levels in maternal plasma, thus implying that increasing NO levels under placental hypoxia may have prevented the exacerbated increase in ischemic placenta-released sFlt-1. Importantly, the present data are consistent with previous reports that demonstrated that NO generation was able to regulate the post-transcriptional processing of mature Flt-1 mRNA, thereby resulting in less of the soluble isoform (sFlt-1) and/or the increased degradation of sFlt-1 mRNA [34,40]. However, mechanism-regulated NO on sFtl-1 transcriptional and post-transcriptional control in preeclampsia need further research.

The placental release of sFlt-1 into the maternal circulation during PE is also related to manifesting systemic vascular dysfunction [41]. In the present study, the dataset demonstrates that increased vascular contraction to the alpha-adrenergic receptor agonist phenylephrine is attenuated in the aorta of pregnant rats submitted to placental ischemia and treated with sodium nitrite. Hence, these results support the idea that nitrite-derived NO may have counteracted vascular hyper-reactivity, which is an important pathophysiological manifestation that underlies increases in maternal blood pressure in pregnancies complicated by PE. Moreover, our results suggest that sodium nitrite-derived NO protected endothelium and restored acetylcholine-induced relaxation in the aortas of pregnant rats submitted to placental ischemia and treated with sodium nitrite. To further confirm that the vascular muscle layer is responsive to NO independently of endothelium, additional vascular reactivity experiments were carried out to examine the exogenous NO donor (sodium nitroprusside)-induced responses. Our findings show no significant difference on sodium nitroprusside-induced vascular relaxation in aortas (with and without endothelium) in all experimental groups. Thus, our results suggest that exogenous NO, including sodium nitrite-derived NO, may be an alternative to restoring the NO and producing vascular relaxation independently of the endothelium. Consistent with the present study, prior evidence has shown that the same sodium nitrite dosage that was chosen for our current study also reduced the blood pressure in pregnant rats treated with a non-selective NO synthase inhibitor (L-NAME) [16], which is in accordance with the hypothesis that the antihypertensive action of sodium nitrite may be independent of endogenous NO production by the endothelium.

The protective effects of sodium nitrite treatment on the maternal–fetal interface of preeclamptic rats may be explained by the fact that nitrite-derived NO restored the blood flow necessary for fetal and placental development in the RUPP+Na-Nitrite rats. However, the present results demonstrate concern related to the sodium nitrite effects in the Preg+Na-Nitrite rats once significant reductions were found in the measurements of maternal blood pressure, which could have also reduced blood perfusion to the uteroplacental vasculature, and it has impaired fetal and placental development in the Preg+Na-Nitrite group. Moreover, in the present study, aortas were used in vascular function experiments because there is previous evidence demonstrating that increased hemodynamic stress and structural changes of the vascular wall contribute to the aorta dilatation, dissection, and rupture in pregnancy [42], which can be further severe in aortas of hypertensive pregnancy [42,43]. However, once conductance vessel aorta, but not resistance vessels, was used in the present study, further investigation is needed to confirm our results.

## 4. Materials and Methods

### 4.1. Animals

The experimental design was approved by the Institutional Animal Care and Use Committee (protocols no. 7946200721 and no. 6707090320) of the Institute of Biosciences, São Paulo State University Julio de Mesquita Filho. All procedures were performed in accordance with the guidelines of Animal Research: Reporting of In Vivo Experiments (ARRIVE) guidelines.

Female Wistar rats were housed in cages and placed in a vivarium with a 12 h light/dark cycle at a temperature of 22 ± 2 °C, with unlimited access to water and food. All animals were acclimatized before the mating process. The mating process occurred with the insertion of one male and two females rats by cage (Harem system) during the night. The next day, vaginal smear was performed, and the presence of sperm and estrus cells was determined as pregnancy day 1.

### 4.2. RUPP Model of PE and Experimental Protocol in Pregnant Rats

On pregnancy day 14, pregnant rats from RUPP and RUPP+Na-Nitrite groups were submitted to the RUPP (reduced uteroplacental perfusion pressure) surgical model of PE, as previously described [44,45,46]. Briefly, three silver clips were inserted as follows: one clip (0.203 mm, internal diameter) was placed into the lower abdominal aorta (above the iliac bifurcation), and the other two clips (0.100 mm, internal diameter) were placed into the right and left branches of each ovarian artery with the aim of reducing the blood perfusion to the uteroplacental circulation. Pregnant rats from Preg and Preg+Na-Nitrite groups were submitted to the Sham surgery on pregnancy day 14, in which similar abdominal incision and suturing without clip placement were performed.

Pregnant rats were randomly divided into four experimental groups (*n* = 8–10 animals per group): pregnant rats treated with saline (Preg group), pregnant rats treated with sodium nitrite (Preg+Na-Nitrite group), pregnant rats submitted to the RUPP model of PE and treated with saline (RUPP group), and pregnant rats submitted to the RUPP model of PE and treated with sodium nitrite (RUPP+Na-Nitrite).

The dose of sodium nitrite (S2252, Sigma, St. Louis, MO, USA) of 15 mg/kg/day (equivalent to 0.217 mmol/kg) was administered via gavage (once a day) in animals from Preg+Na-Nitrite and RUPP+Na-Nitrite groups. Previous studies have shown that this dose of sodium nitrite has antihypertensive effects in rats [47,48]. Animals from Preg and RUPP groups received saline solution administered via gavage (once a day). Sodium nitrite or saline was administered from pregnancy days 14 to 21.

### 4.3. Maternal Blood Pressure Measurements

Maternal blood pressure was recorded on pregnancy days 11 and 13 using the non-invasive method of tail plethysmography (Insight, Ribeirão Preto, São Paulo, Brazil, catalog #EFF-306). Briefly, rats were placed in a heated box (Insight, Ribeirao Preto, Sao Paulo, Brazil, catalog # EFF-307) for 10 min and were conditioned to three cycles of cuff inflation and deflation by a trained operator for the plethysmography measurements. The recordings were performed to confirm that all pregnant rats had similar basal blood pressure values before RUPP or Sham procedures.

On pregnancy day 21, all animals were anesthetized with isoflurane (2%; Cristalia, São Paulo, Brazil), and a polyethylene catheter (PE-50 # 2,270,835, Thermo Fisher Scientific, Waltham, MA, USA) was implanted in the left carotid artery, connected to a pressure transducer attached to an amplifier and pressure recorder of data acquisition system (MP150CE, Biopac Systems Inc., Goleta, CA, USA). Systolic blood pressure (SBP) was measured in anesthetized animals, and recorded by AcqKnowledge software (Version 3.2.1) using a data acquisition system (Biopac Systems Inc., Goleta, CA, USA).

### 4.4. Euthanasia, Blood Sampling, and Tissue Harvest

After blood pressure measurements on pregnancy day 21 (the last day of the experimental protocol), the animals were euthanized by an overdose of isoflurane (5%; Cristalia, São Paulo, Brazil) followed by cardiac puncture in the left ventricle to collect blood. Blood sample was placed in lyophilized heparin-containing tube (Vacutainer, Becton Dickinson, Oxford, UK) and was centrifuged at 10.000 rpm (for 10 min) for plasma separation. Plasma samples were stored at −80 °C for biochemical analysis. The thoracic aorta was surgically removed and placed in a cold Krebs physiological solution for vascular function experiments. An incision was made in the abdomen, the uterus was excised, and cesarean section was performed to examine fetuses and placentas.

### 4.5. Fetal and Placental Parameters

Litter size (total number of pups) was recorded. Each fetus was removed from the amniotic sac and separated from its respective placenta. The weight of each pup and the placental weights were also recorded. Values related to weights were expressed in grams (g).

### 4.6. Vascular Reactivity Experiments

Vascular contraction and relaxation responses were carried out as previously described [27]. The thoracic aorta was carefully cleaned with the aid of a dissection microscope (SZO-T, Optika Microscopes, Ponteranica, BG, Italy). Thoracic aorta segments (3–4 mm wide rings) were cut, and endothelium was preserved intact in two rings, whereas endothelium was mechanically removed from the other two rings by scraping the thoracic aortic ring interior five times around the tip of forceps. Endothelium-denuded rings were discarded if there was any degree of relaxation. Each thoracic aortic ring was suspended between two wire hooks. One hook was fixed and immersed at the bottom of the organ chamber containing 10 mL of Krebs–Henseleit solution (composition in mmol/L: NaCl 130; KCl 4.7; CaCl_2_ 1.6; KH_2_PO_4_ 1.2; MgSO_4_ 1.2, NaHCO_3_ 15; glucose 11.1), and the other hook was attached to an isometric amplified force transducer (FORT10, Transbridge 4M, World Precision Instruments, WPI, Sarasota, FL, USA). Tension-induced changes in all thoracic aortic rings were recorded and analyzed using AcqKnowledge software (version 3.5.7, MP100, Biopac Systems Inc., Goleta, CA, USA). Thoracic aortic rings were stretched under 1.5 g basal tension and were allowed to equilibrate for 45 min in temperature-controlled (37 °C) organ chambers filled with 10 mL of Krebs–Henseleit solution, bubbled with a mixture of 95% O_2_/5% CO_2_, and maintained at pH 7.4. Krebs–Henseleit solution was changed every 15 min. After thoracic aortic ring equilibration, control contraction to potassium chloride (KCl, 96 mM; P5405, Sigma, St. Louis, MO, USA) was elicited. Once maximal KCl-induced contraction was observed, the thoracic aortic rings were rinsed (3 times) with Krebs–Henseleit solution, 10 min each. The resting tension in all thoracic aortic rings showed no difference.

To determine the contractile activity of vascular smooth muscle, endothelium-intact and endothelium-denuded thoracic aortic rings were stimulated with increasing concentrations (10^−10^ to 10^−4^ M) of phenylephrine (P6126, Sigma, St. Louis, MO, USA). After that, each thoracic aortic ring was sequentially rinsed (3–5 times) with Krebs–Henseleit solution and was allowed to relax to baseline. Thirty minutes later, endothelium-intact and endothelium-denuded thoracic aortic rings were pre-contracted with phenylephrine (10^−6^ M), and after reaching a stable and sustainable contraction, increasing concentrations (10^−9^ to 10^−4^ M) of acetylcholine (A6625, Sigma, St. Louis, MO, USA) were added to the organ chamber to investigate endothelium-dependent vasodilation.

To examine the contribution of endothelium-derived NO-dependent relaxation, a 30 min pre-incubation with a non-selective inhibitor of NO syntheses (Nωnitro-L-arginine methyl ester, L-NAME, 3 × 10^−4^ M; N5751, Sigma, St. Louis, MO, USA) was performed, and increasing concentrations (10^−9^ to 10^−4^ M) of acetylcholine were added to the organ chambers containing the endothelium-intact thoracic aortic rings.

To investigate the ability of vascular smooth muscle to respond to NO, increasing concentrations (10^−9^ to 10^−6^ M) of exogenous NO donor (sodium nitroprusside, SNP; 71778, Sigma, St. Louis, MO, USA) were added to the organ chambers, in which endothelium-intact and endothelium-removed thoracic aortic rings had been pre-contracted with phenylephrine (10^−6^ M) and had reached a stable and sustainable contraction.

Individual concentration–contraction and concentration–relaxation response curves were analyzed using nonlinear regression, and sigmoidal dose–response curves were fitted using the least squares method. The effective concentration that produced half the maximal response (EC_50_) was calculated and expressed as pEC_50_ (−log M). The concentration–contraction curves to phenylephrine were expressed in g, and concentration–response curves to acetylcholine (with or without endothelium and/or L-NAME) and sodium nitroprusside (with or without endothelium) were expressed as the % relaxation to phenylephrine-induced contraction, as previously described [44].

### 4.7. Determination of NO Metabolites (NOx) in Plasma

To quantify NOx in plasma, Griess reagent was used, as previously described [49]. Plasma samples (190 μL) were deproteinized with 10 μL of zinc acetate solution (300 g/L) followed by 30 min centrifugation (10,000× *g*, at 4 °C). After that, microplate shaker (Eppendorf Thermo-Mixer, FP, Darmstadt, Germany) was used to the controlled-agitation and 30 min incubation (at 37 °C and in the absence of light), in which plasma supernatant (50 μL) was pipetted into wells containing 50 μL of sulfanilamide (2%, *m*/*v*; S9251, Sigma, St. Louis, MO, USA); 50 μL of 0.1% *n*-(1-naphthyl)-ethylenediamine dihydrochloride, NED (*v*/*v*; N9125, Sigma, St. Louis, MO, USA); and 100 μL of vanadium chloride III (5%, *m*/*v*; 208272, Sigma, St. Louis, MO, USA) solution. Prepared nitrite solutions with deionized water and Griess reagents were used to make the calibration curve (1.56–100 μmol/L). Absorbance was read at 535 nm in a spectrophotometer (Synergy 4, Biotek, Winooski, VT, USA). NOx levels in plasma were expressed as μmol/L.

### 4.8. Determination of Plasma Levels of sFlt-1

A commercial enzyme-linked immunoassay (ELISA) kit for sFlt-1 (MBS2602003; MyBioSource, San Diego, CA, USA) was used. The assay was carried out according to the manufacturer′s instructions. Plasma levels of sFlt-1 were expressed as pg/mL.

### 4.9. Statistical Analysis

GraphPad Prism^®^ software (version 8.0; San Diego, CA, USA) was used for result analysis. The normality of data distribution was verified by Shapiro–Wilk tests. One-way analysis of variance (ANOVA) and repeated-measure two-way ANOVA followed by Tukey post hoc tests were applied for multiple comparisons. A value of probability (*p*) < 0.05 was considered statistically significant. The results are expressed as mean ± standard error of the mean (SEM).

## 5. Conclusions

The present results demonstrate that sodium nitrite-derived NO may be an alternative to restore circulating NO levels and thus attenuate the increases in sFlt-1 levels in maternal plasma in a placental ischemia-induced model of PE. Our findings also suggest that increases in maternal blood pressure as well as vascular and endothelial dysfunctions are mitigated by sodium nitrite treatment, implying that nitrite-derived NO may be involved.

## Data Availability

The authors declare that all the data supporting the results of the present study are included in the article.

## Supplementary Materials

The following supporting information can be downloaded at https://www.mdpi.com/article/10.3390/ijms252011051/s1
