# Supplementary material for: Impaired Endothelium-Dependent Vasodilation and Increased Levels of Soluble Fms-like Tyrosine Kinase-1 Induced by Reduced Uterine Perfusion Pressure in Pregnant Rats: Evidence of Protective Effects with Sodium Nitrite Treatment in Preeclampsia
Source: Int J Mol Sci. 2024 Oct 15;25(20):11051. doi: 10.3390/ijms252011051 (PMC11507509; doi:10.3390/ijms252011051)
Supplement: Supplementary file 1 [file ijms-25-11051-s001.zip › ijms-3251591-supplementary.pdf]

Table S1: Maximal response (E_{max}) and negative logarithm of the concentration that evoked 50% of the maximal response (pEC_{50}) recorded for phenylephrine, acetylcholine, and sodium nitroprusside in aortic rings with (n = 2 rings per animal) and without (n = 2 rings per animal) endothelium from Preg, Preg+Na-Nitrite, RUPP, and RUPP+Na-Nitrite groups (n = 8-10 animals per group).

Parameters	Group			
	**Preg**	**Preg+Na-Nitrite**	**RUPP**	**RUPP+Na-Nitrite**
**Intact-aortic rings**				
Phenylephrine E_{max} (g)	2.04 ± 0.09	2.17 ± 0.08	2.85 ± 0.13*	2.16 ± 0.11
Phenylephrine pEC_{50} (−log M)	7.02 ± 0.10	6.83 ± 0.16	8.01 ± 0.26	6.83 ± 0.33
**Denuded-aortic rings**				
Phenylephrine E_{max} (g)	2.53 ± 0.19	2.17 ± 0.10	3.33 ± 0.45*	2.49 ± 0.15
Phenylephrine pEC_{50} (−log M)	6.61 ± 0.10	6.50 ± 0.21	7.09 ± 0.22	6.86 ± 0.33
**Intact-aortic rings**				
Acetylcholine E_{max} (%)	91 ± 16	85 ± 2*	64 ± 9*	73 ± 3*
Acetylcholine pEC_{50} (−log M)	6.23 ± 0.17	7.08 ± 0.51	5.04 ± 0.44	7.63 ± 1.34
Acetylcholine + L-NAME E_{max} (%)	4 ± 1	6 ± 3	6 ± 2	8 ±2
**Intact-aortic rings**				
Sodium nitroprusside E_{max} (%)	97 ± 2	99 ± 1	99 ± 1	98 ± 1
Sodium nitroprusside pEC_{50} (−log M)	10.14 ± 1.01	9.07 ± 0.30	9.10 ± 0.18	9.03 ± 0.64
**Denuded-aortic rings**				
Sodium nitroprusside E_{max} (%) in	99 ± 1	99 ± 1	99 ± 1	96 ± 1
Sodium nitroprusside pEC_{50} (−log M)	10.87 ± 1.23	8.92 ± 0.63	9.46 ± 0.65	9.46 ± 0.93

Data are expressed as means ± SEM. *$p < 0.05$ vs Preg group.
